# Antimicrobial Efficacy of Chitosan vs. Sodium Hypochlorite Against *Enterococcus faecalis*: A Systematic Review and Meta-Analysis

**DOI:** 10.3390/medicina62081491

**Published:** 2026-08-03

**Authors:** Virág Róna, Bulcsú Bencze, Gergely Agócs, Péter Hegyi, Kata Kelemen, Tamás Hegedüs, Gábor Varga, Beáta Kerémi, Adél Galvács, Zoltán Géczi

**Affiliations:** 1 Department of Prosthodontics, Semmelweis University, 1085 Budapest, Hungary; rona.virag@semmelweis.hu (V.R.);; 2 Centre for Translational Medicine, Semmelweis University, 1085 Budapest, Hungary; 3 Department of Biophysics and Radiation Biology, Semmelweis University, 1085 Budapest, Hungary; 4 Institute of Pancreatic Diseases, Semmelweis University, 1083 Budapest, Hungary; 5 Institute for Translational Medicine, Medical School, University of Pécs, 7624 Pécs, Hungary; 6 Department of Oral Biology, Semmelweis University, 1089 Budapest, Hungary; 7 Department of Restorative Dentistry and Endodontics, Semmelweis University, 1088 Budapest, Hungary

**Keywords:** chitosan, sodium hypochlorite, periapical, root canal, enterococcus faecalis, *E. faecalis*, irrigation

## Abstract

*Background and Objectives*: This study compared the antimicrobial effects of chitosan and sodium hypochlorite against *Enterococcus faecalis* (*E. faecalis*) at different irrigation solution concentrations. *E. faecalis* is a persistent pathogen frequently surviving root canal treatment, while sodium hypochlorite remains the current gold-standard irrigation material. *Materials and Methods*: A systematic review and meta-analysis of in vitro studies was conducted using Cochrane Library, EMBASE, and PubMed. Evidence certainty was assessed with GRADE. Two outcomes were analyzed: colony-forming units (CFU/mL) and the inhibition rate (IR). Two reviewers independently screened studies and extracted data. *Results***:** Of 889 identified records, 10 studies met the inclusion criteria. For CFU/mL, the overall effect was −0.28 (95% CI: −1.54 to 0.97). For IR, chitosan concentrations < 1% showed an effect of –7.35 (95% CI: −27.27 to 12.56), while concentrations ≥ 1% showed an effect of −0.90 (95% CI: −19.24 to 17.45). *Conclusions*: Chitosan demonstrated antimicrobial activity comparable to sodium hypochlorite in vitro, with concentrations above 1% showing enhanced antibacterial effects. However, methodological heterogeneity limits direct comparison, and clinical equivalence cannot be concluded.

## 1. Introduction

Root canal infections are caused by microorganisms, with *Enterococcus faecalis* (*E. faecalis*) being one of the most persistent pathogens. It frequently survives conventional root canal treatment and is a major cause of endodontic failure due to its resistance to intracanal medicaments and irrigants [1,2,3,4,5,6,7].

The average success rate of an endodontic treatment is between 86% and 98%, even when performed under aseptic circumstances in accordance with known therapeutic criteria. Treatment failure is usually due to the persistence of intra-radicular bacteria despite chemo-mechanical cleansing [1]. The virulence of *E. faecalis* may stem from the fact that it can live as a solitary organism in the root canal [2,3,4].

Sodium hypochlorite is the current gold-standard irrigation solution because of its antibacterial activity and ability to dissolve organic tissue. However, its cytotoxicity and limited efficacy against *E. faecalis* have prompted the search for alternative or adjunctive irrigants. EDTA is commonly used alongside sodium hypochlorite to remove the smear layer by dissolving inorganic debris [5,6,7].

Chitosan, a biocompatible and biodegradable polymer derived from chitin, has broad-spectrum antimicrobial and anti-inflammatory properties, making it a promising candidate for endodontic irrigation. Several studies have demonstrated its ability to reduce E. faecalis biofilms and bacterial load while potentially promoting healing through its anti-inflammatory effects [4,8,9,10,11,12].

Advances in biomaterials and infection control may also benefit the management of patients with complex craniofacial disorders requiring multidisciplinary dental care [13].

In addition to its antibacterial properties, chitosan also has an anti-inflammatory effect in the root canal, which can facilitate the healing process. This effect is crucial, as inflammation can contribute to the persistence of *E. faecalis* in the root canal by creating an acidic milieu, which promotes bacterial survival and growth [14].

Despite numerous in vitro studies comparing chitosan and sodium hypochlorite, the available evidence remains inconsistent because of substantial methodological variability. At present, no quantitative synthesis has specifically evaluated whether chitosan provides antimicrobial efficacy comparable to sodium hypochlorite against Enterococcus faecalis. Therefore, the aim of the present systematic review and meta-analysis was to compare the effect of chitosan and sodium hypochlorite against *E. faecalis*, with different irrigation solution concentrations.

Our colleagues have investigated several dental applications of polycationic polymers, demonstrating their potential in biomedical innovation. A Trojan-horse drug delivery system has shown promise for targeted periodontitis treatment, while the intrinsic fluorescence of poly(ethyleneimine) (PEI) highlights its potential in imaging and drug delivery [15,16].

In addition, an antimicrobial silver–polyethyleneimine–polylactic acid composite film has been developed for denture coatings to reduce microbial infections [17].

Last year, we also conducted a meta-analysis on the effect of chitosan on Streptococcus mutans, the primary bacterium associated with dental caries [18].

The aim of this study was to compare the effect of chitosan and sodium hypochlorite against *E. faecalis*, with different irrigation solution concentrations.

It is hypothesized that chitosan works more effectively than sodium hypochlorite at reducing the amount of *E. faecalis* in the root canal.

## 2. Materials and Methods

The results of our meta-analysis and systematic review are presented in compliance with PRISMA 2020 criteria and the Cochrane Handbook (Supplementary Table S5) [19]. PROSPERO received the research protocol, which was registered under registration number CRD42023385163 (registration date: 19 December 2022).

### 2.1. Eligibility Criteria

Controlled in vitro studies evaluating the antimicrobial efficacy of chitosan-based irrigants against Enterococcus faecalis were eligible for inclusion. Studies were required to include sodium hypochlorite as the comparator and to report quantitative antibacterial outcomes, including colony-forming units (CFU/mL) or inhibition rate (IR). Only studies using standardized E. faecalis strains and providing sufficient quantitative data for meta-analysis were included. Reviews, case reports, conference abstracts, animal studies, clinical studies, and articles not published in English were excluded.

### 2.2. Information Sources

The initial search was conducted on 1 December 2022 and updated on 10 July 2023. The search was restricted to studies published between 2016 and 2023 to reflect contemporary evidence regarding the use of chitosan as an endodontic irrigation material. The following search key was used: (root canal OR root canal disinfection OR endodontic) AND (irrigation OR chitosan OR chitosan nano particles OR quaternary chitosan OR derivative OR sodium OR hypochlorite OR sodium hypochlorite OR sodium-hypochlorite) AND (antimicrobial efficacy OR antibacterial activity OR antibacterial effect OR antimicrobial mechanism) AND (enterococcus faecalis OR e. faecalis OR enterococcus). We applied a time filter between 2016 and 2023 due to the greater material use in dentistry.

### 2.3. Selection Process and Data Collection

Three databases were searched for articles: PubMed, EMBASE, and Cochrane Library. Following the elimination of duplicates, two independent review writers (Author A and Author B) chose the complete text by looking at the title and abstract. Two writers (Author A and Author B) separately gathered data from relevant publications. Any differences in opinion were settled by a third author (Author C). The following information was extracted: first author, publication year, DOI, study type, study population, sample size in the intervention group, and sample size in the comparator group.

### 2.4. Study Risk of Bias Assessment

Two authors (Author A and Author B) independently performed a risk of bias assessment using the Risk of Bias Tool 2 (ROB2) (Supplementary Table S3). We also included the QUIN tool in the Supplementary Materials (Supplementary Table S4). Researchers can utilize the QUIN tool to assess the potential for bias in specific in vitro investigations. This tool offers a consistent methodology for assessing the risk of bias in in vitro studies [20].

Quality and certainty assessments of the studies included were performed according to the GRADE handbook (Supplementary Table S2) using the GRADE-PRO website (https://www.gradepro.org/). Assessments were performed independently by two authors (Writer A and Writer B). In cases of disagreement, a third author was involved (Author C).

### 2.5. Synthesis Methods

Microsoft Excel was used to extract and arrange primary data. The statistical analysis was performed in R (version 4.1.3, 3 October 2022) [21]. The metacont function of the meta R package (version 6.1.0) was used, and we pooled the inhibition rate as the mean difference (chitosan arm—hypochlorite arm) [22]. To account for inherent variability and heterogeneity within individual studies, a random effects method was adopted. The constrained maximum-likelihood estimator was used to calculate between-study variance tau squared. [23] We used Knapp–Hartung adjustments to estimate the standard error [24,25].

## 3. Results

### 3.1. Study Selection

**Figure 1:**
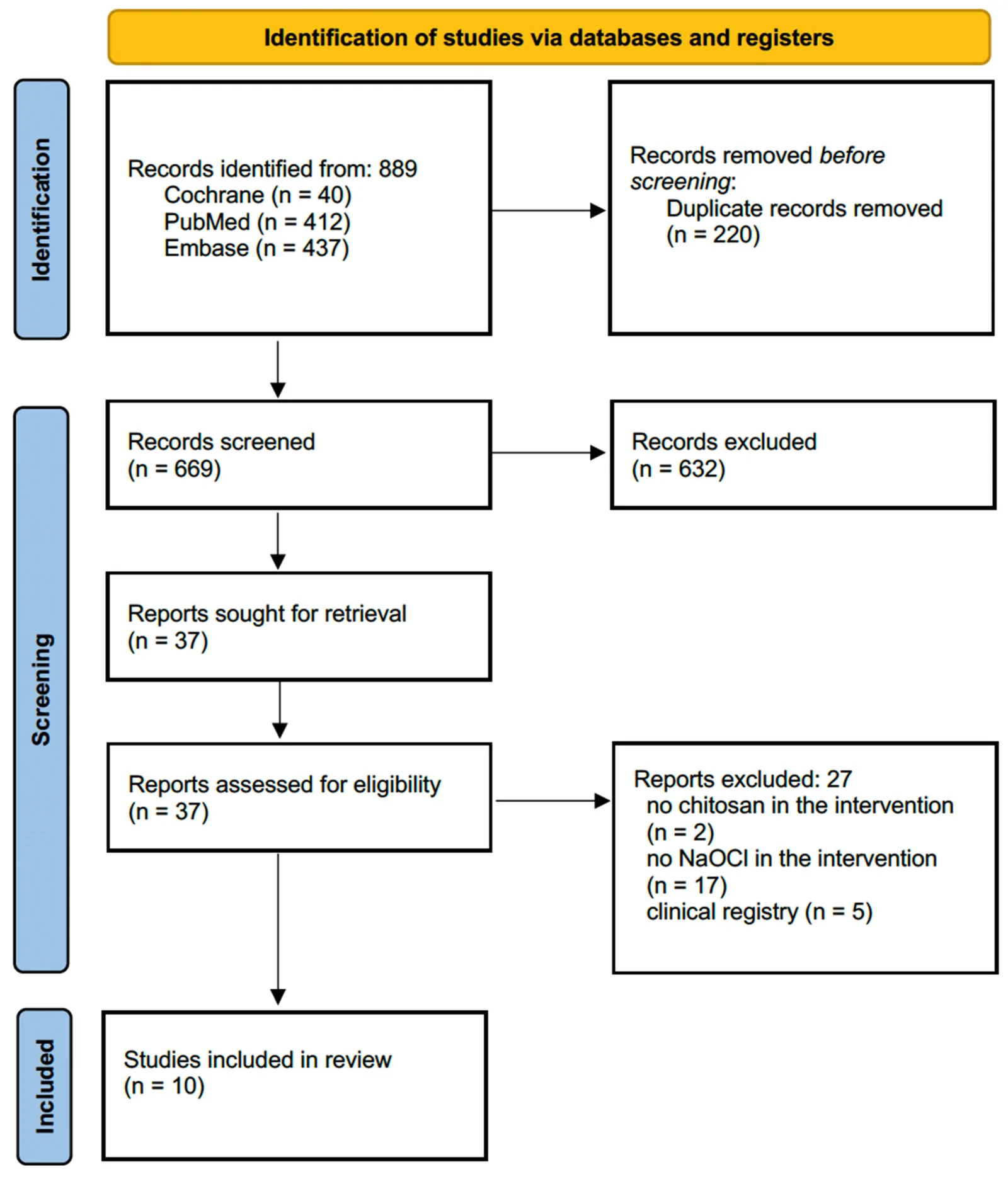
PRISMA flowchart.

A total of 889 articles were downloaded from three databases: EMBASE (*n* = 437), PubMed (*n* = 412), and Cochrane Library (*n* = 40) (Figure 1).

After duplicate removal, 669 articles remained. For full-text selection, 37 abstracts were selected.

After the selection process, a total of 10 articles were included in the qualitative and quantitative synthesis. The full selection process can be seen in the Prisma flowchart (Figure 1).

### 3.2. Characteristics of the Studies Included

The main characteristics of the studies included in the analysis are detailed in Supplementary Table S1. All studies were in vitro studies.

### 3.3. Results of Individual Studies and the Synthesis

#### 3.3.1. The Number of *Enterococcus faecalis* in CFU/mL

**Figure 2:**
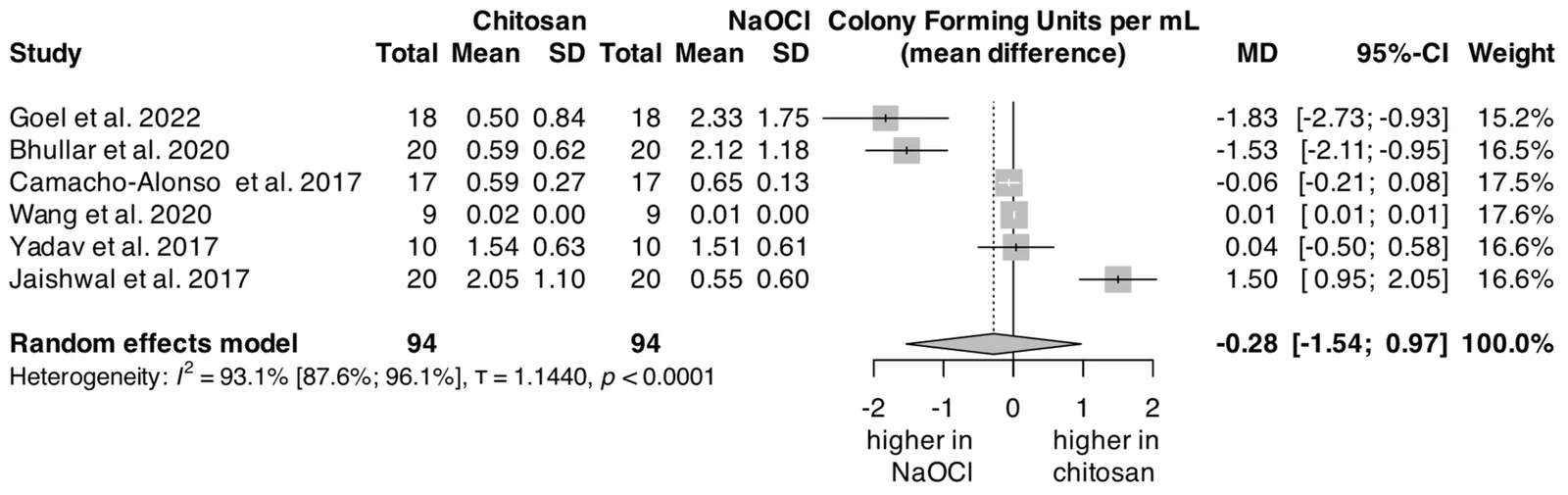
The number of Enterococcus faecalis in CFU/mL (mean difference) [4,9,11,26,27,28].

Six articles were included in this analysis [4,9,11,26,27,28] (Figure 2). The overall effect was −0.28, as measured by the mean difference, indicating a trend toward chitosan. The confidence interval was [−1.54, 0.97]. Although this result is not mathematically significant, it is clinically relevant, as we can say that the difference in the antibacterial effect between chitosan and sodium hypochlorite was negligible. Three articles found no significant differences [4,9,28] and another three publications found significant differences [11,26,27]. Goel and Bhullar showed that chitosan is significantly better than sodium hypochlorite. The mean difference in the article by Goel et al. was −1.83, and the confidence interval was [−2.73, −0.93]. The mean difference in the study by Bhullar et al. was −1.53, and the CI was [−2.11, −0.95]. The I^2^ value was 93%, showing high heterogeneity. The paper of Jaishwal et al. [27] stated that sodium hypochlorite had better effects. While the figure indicates a positive mean difference in this case the trend of sodium hypochlorite outperforming chitosan is not consistent across the other studies. On the other hand, Bhullar et al. [11], who used exactly the same method, concluded that chitosan had a greater antibacterial effect. As two articles in the forest plot showed that chitosan was favored and three articles found no significant difference between the antibacterial effect of the two investigated materials, it seems to be likely that chitosan is as good as sodium hypochlorite or in some cases even better. However, since the confidence interval includes zero, the result does not statistically favor either chitosan or sodium hypochlorite.

#### 3.3.2. The Number of *Enterococcus faecalis*—Inhibition Rate (IR)

**Figure 3:**
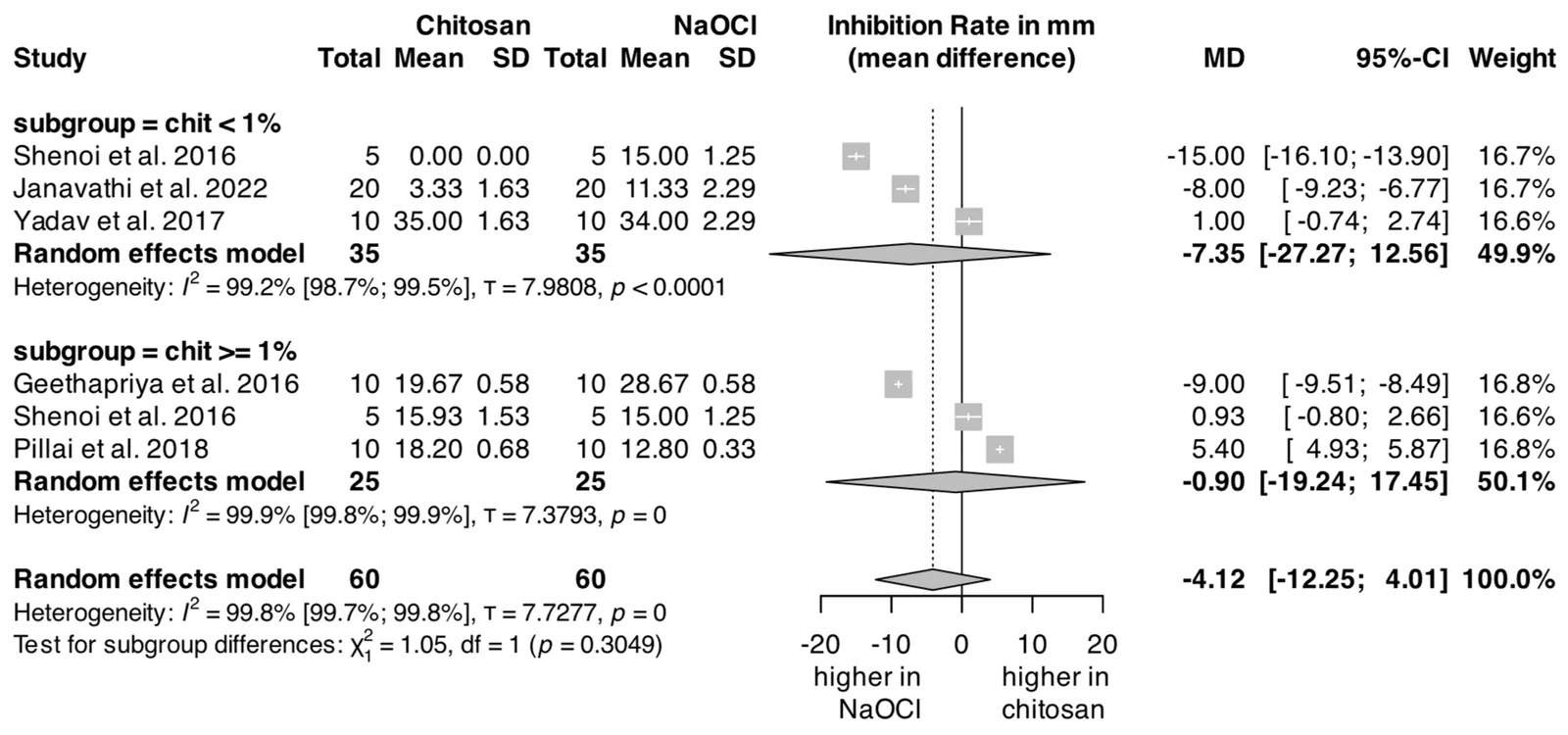
The number of Enterococcus faecalis in terms of inhibition rate—mm (mean difference) [10,12,28,29,30].

Five studies were included and [10,12,28,29,30] two subgroups were used for this analysis (Figure 3). Chitosan concentration was below 1% in the first subgroup, and chitosan was equal or above 1% in the second group. In the first subgroup, the overall effect was −7.35, as measured by the mean difference. The confidence interval was [−27.27, 12.56]. In the second subgroup, the overall effect was −0.90, as measured by the mean difference. The confidence interval was [−19.24, 17.45]. The I^2^ value was 100%.

Chitosan becomes as effective as sodium hypochlorite with increasing concentrations. The articles employed escalating chitosan concentrations alongside increasing levels of sodium hypochlorite.

### 3.4. Risk of Bias in Studies and Certainty of Evidence

The ROB2 tool was used for the risk of bias analysis. The methodological assessment suggested a generally low risk of bias across the evaluated domains. Nevertheless, these findings should be interpreted cautiously because the included studies were exclusively laboratory-based in vitro investigations, and methodological quality does not necessarily translate into clinical applicability (Supplementary Table S3).

Certainty of evidence was assessed using the GRADE-PRO tool (Supplementary Table S2). Although the assessment suggested a high certainty for the evaluated outcome, this rating should be interpreted with caution because the available evidence was derived exclusively from in vitro studies. Consequently, the certainty reflects the internal consistency of the included experimental studies rather than the certainty of clinical effectiveness.

## 4. Discussion

The present meta-analysis demonstrated that chitosan exhibited antimicrobial activity comparable to sodium hypochlorite against Enterococcus faecalis under the investigated in vitro conditions. However, these findings should be interpreted with caution. Comparable pooled effect estimates do not constitute evidence of clinical equivalence, as no formal equivalence or non-inferiority analysis was performed, and all included studies were laboratory-based. Furthermore, substantial methodological heterogeneity among the included studies, including differences in chitosan formulation, concentration, exposure time, and experimental protocols, limits the direct translation of these findings into clinical practice.

The present meta-analysis demonstrated no mathematical significance in the antimicrobial efficacy of chitosan and sodium hypochlorite against Enterococcus faecalis under in vitro conditions. Although these findings suggest comparable antibacterial performance, considerable methodological heterogeneity (Table 1) among the included studies should be considered when interpreting the pooled estimates.

An important limitation of the available evidence is that most studies failed to report key physicochemical characteristics of chitosan, including molecular weight and degree of deacetylation. Both parameters directly influence the cationic charge density, solubility, and antimicrobial activity of chitosan.

The concentration of chitosan varied across the included studies, ranging from 0.2% to 2%. Such variability may have influenced antimicrobial performance, as an increasing chitosan concentration provides a greater density of protonated amino groups capable of interacting with negatively charged bacterial cell walls, thereby enhancing membrane disruption. Therefore, we used two subgroups where the chitosan concentration was below 1% in the first one, and chitosan was equal to or above 1% in the second one.

Only a minority of the included studies reported the molecular weight of the chitosan preparation. This parameter has been shown to influence biofilm penetration and antibacterial activity. The lack of standardized reporting limits comparisons among studies and may represent an unrecognized source of heterogeneity.

Similarly, the degree of deacetylation was rarely reported despite being one of the principal determinants of chitosan cationic charge density and antimicrobial activity.

Exposure time also differed considerably among the included investigations. Longer contact times may increase bacterial membrane disruption and biofilm penetration, thereby enhancing antibacterial efficacy.

Overall, the first outcome revealed that chitosan can be superior to or as good as sodium hypochlorite. The second outcome showed similar results when chitosan was used at concentrations above 1%. Although our findings were not statistically significant, they may have clinically meaningful implications.

A major limitation of the available evidence is the inconsistent reporting of key physicochemical characteristics of chitosan. Molecular weight was clearly reported in only one study and qualitatively described in another, while the degree of deacetylation or substitution was absent in most studies. Since molecular weight and deacetylation degree influence solubility, cationic charge density, biofilm penetration, and interaction with bacterial cell walls, the lack of standardized reporting limits direct comparison among studies.

Sodium hypochlorite (NaOCl) is the gold-standard root canal irrigant due to its strong antibacterial activity and ability to dissolve organic tissue. It eliminates bacteria, including E. faecalis, through oxidative and chlorination reactions. In aqueous solution, NaOCl forms hypochlorous acid (HOCl), which penetrates bacterial cells, denatures proteins, disrupts membranes, and damages DNA, leading to rapid cell death. Its antimicrobial efficacy depends on concentration, contact time, pH, and temperature. However, high concentrations are cytotoxic and may cause tissue damage and postoperative sensitivity [31,32,33,34,35].

As a result, various irrigants, including chitosan, were investigated for antibacterial effectiveness against *E. faecalis*. It is effective against a wide range of Gram-negative and Gram-positive bacteria [36].

Chitosan has therefore emerged as a promising alternative because of its broad-spectrum antibacterial activity, excellent biocompatibility, and minimal cytotoxicity at therapeutic concentrations. Its positively charged amino groups interact with negatively charged bacterial cell walls, increasing membrane permeability and causing leakage of intracellular components. Low-molecular-weight chitosan can also penetrate bacterial cells and interfere with nucleic acid synthesis. Its antimicrobial activity is influenced by pH, molecular weight, degree of deacetylation, concentration, and ionic composition [37,38].

Chitosan exerts antibacterial activity through multiple mechanisms. Its polycationic amino groups bind to negatively charged bacterial cell walls, disrupting membrane integrity, increasing permeability, and causing leakage of intracellular components, ultimately leading to cell death. Low-molecular-weight chitosan can also penetrate bacterial cells and interfere with nucleic acid synthesis, inhibiting transcription and translation. Its antimicrobial activity is influenced by pH, molecular weight, degree of deacetylation, concentration, and ionic composition. Acidic conditions, a low molecular weight, and a high degree of deacetylation enhance its efficacy, whereas high Ca^2+^ and Mg^2+^ concentrations may reduce antibacterial activity. Higher chitosan concentrations generally produce stronger bactericidal effects [39].

The low possibility for adverse effects on oral tissues due to the biocompatibility and biodegradability of chitosan makes it a good alternative for these uses, which is important for avoiding postoperative complaints. However, the molecular weight, concentration, and composition of chitosan may all play a role in its effectiveness against *E. faecalis* [40,41,42,43]. The usage of chitosan can prevent a significant number of postoperative sensitivities that may occur after root canal treatment.

Due to its biocompatibility and biodegradability, chitosan may reduce postoperative complications while maintaining antibacterial efficacy against E. faecalis. In addition, several studies have demonstrated that chitosan effectively removes the smear layer [30,44,45,46,47,48].

Geethapriya et al. showed that combining chitosan with EDTA preserved EDTA’s smear layer removal ability while enhancing antibacterial activity against E. faecalis, with larger inhibition zones observed at higher chitosan concentrations [30].

Silva et al. reported that even 0.2% chitosan removed the smear layer as effectively as 15% EDTA and 10% citric acid [44].

Similarly, Antunes et al. found that final irrigation with 0.2% chitosan produced a smear layer removal and dentin microhardness reduction comparable to 15% EDTA, supporting its potential as an alternative endodontic solution [45].

Methodological heterogeneity was identified across the included studies. Chitosan concentration ranged from 0.2 to 2% in solutions, while some investigations evaluated nanoparticles, HTCC derivatives, chitosan–EDTA combinations, and chitosan–CHX combinations. NaOCl concentrations, contact times, and biofilm maturity also varied considerably. These differences may influence bacterial killing of the active irrigation independently and should be considered when interpreting the pooled estimates.

**Figure 4:**
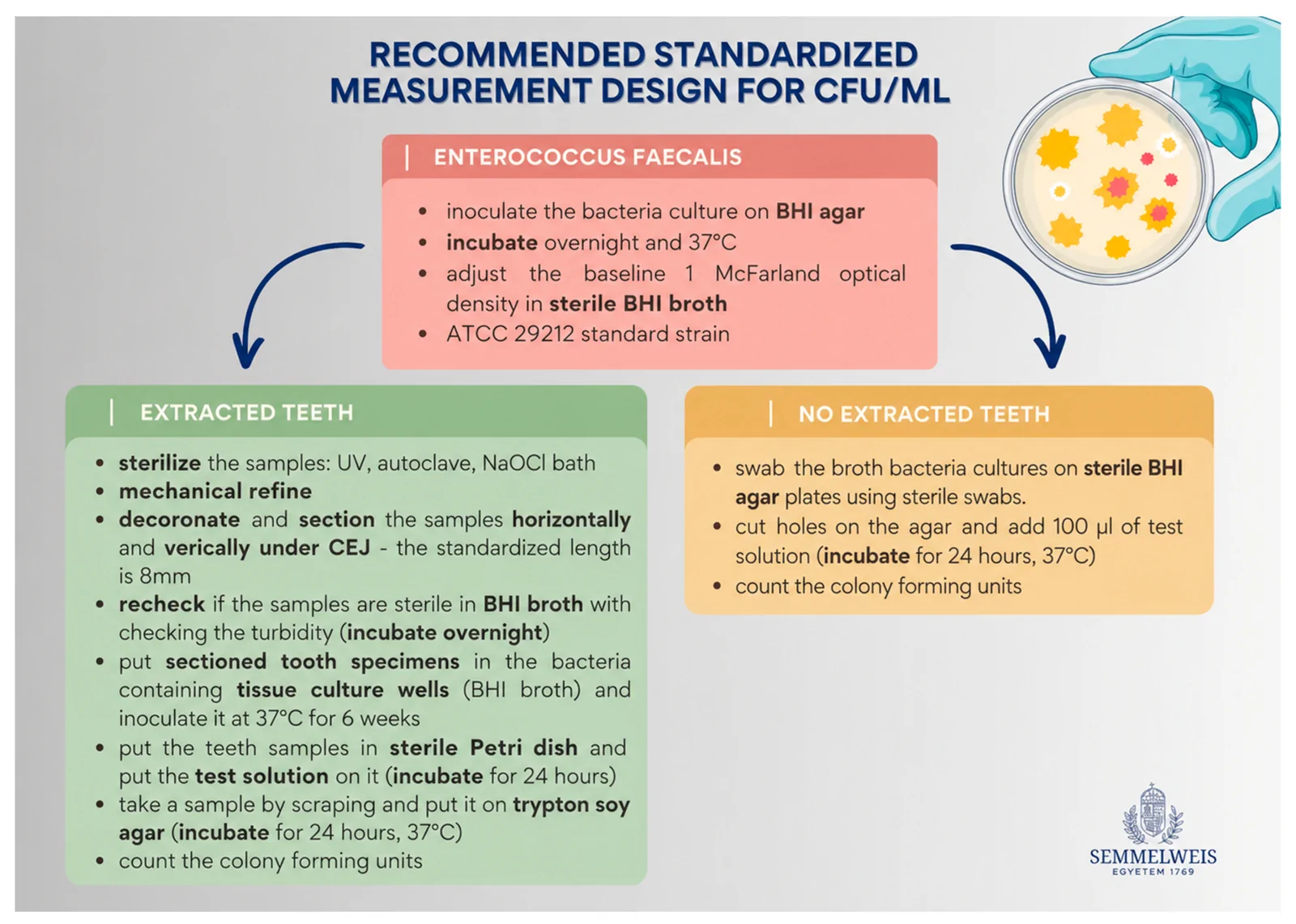
The recommended standardized measurement design for future studies—CFU/mL.

**Figure 5:**
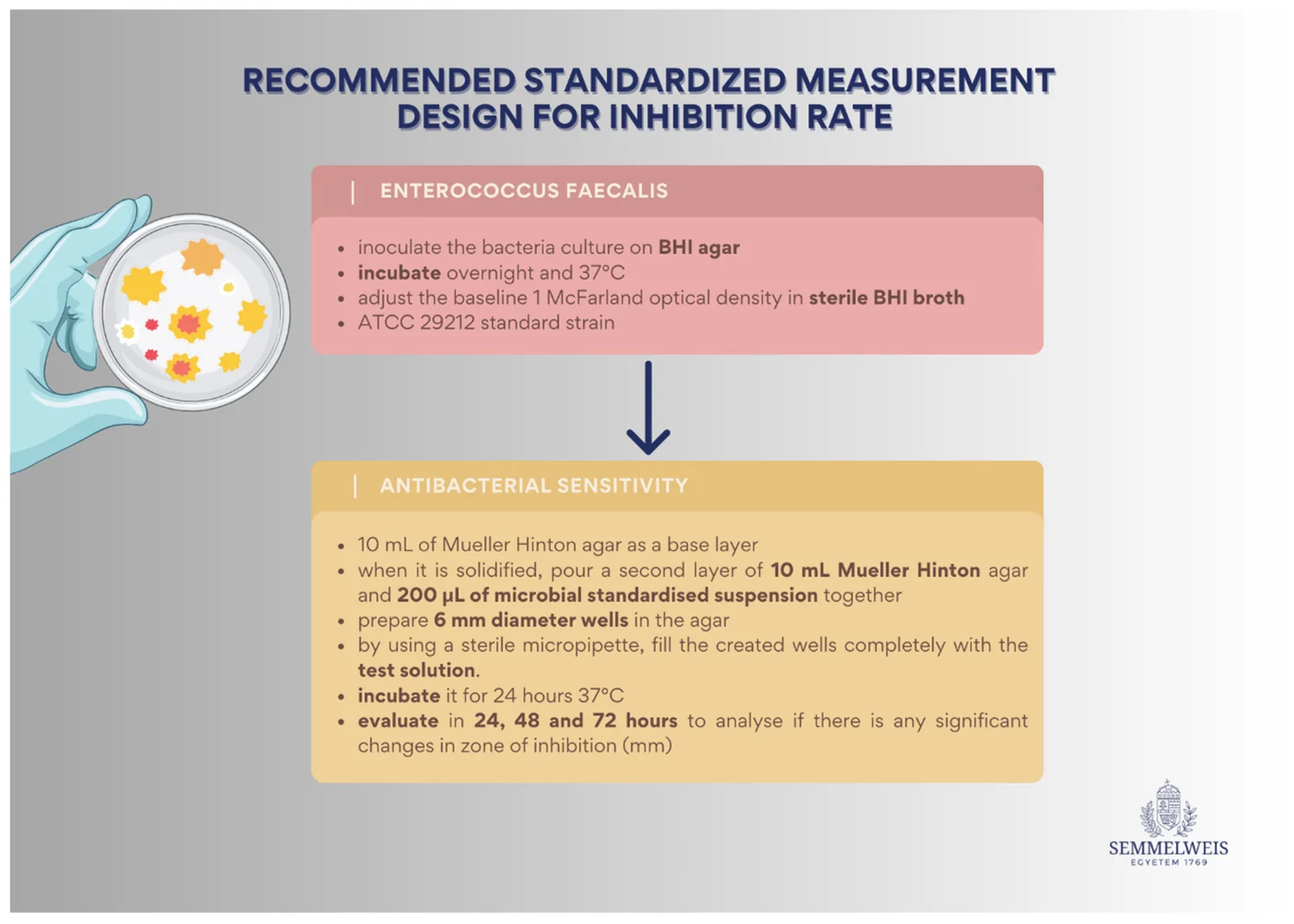
The recommended standardized measurement design for future studies—IR.

The findings of the studies are diverse, which might be due to the lack of a consistent test protocol. The study methods were not completely consistent across all publications; we therefore recommend that a standardized measurement design should be used. This proposed design is shown in Figure 4 and Figure 5.

The ideal concentration of sodium hypochlorite is higher than 1% (2.5%). It is also less cytotoxic than the 5.25% concentration. In 5 min, 2.5% sodium hypochlorite may inhibit 100% of *E. faecalis* [49].

As indicated in the second outcome, sodium hypochlorite was more efficient than chitosan when the concentration of chitosan was less than 1%, but when the concentration was greater than 1%, chitosan appeared as excellent as, if not better than, hypochlorite.

In other words, the available in vitro evidence suggests that chitosan demonstrated antimicrobial activity comparable to that of sodium hypochlorite against E. faecalis. However, these findings should not be interpreted as evidence of clinical equivalence given the methodological heterogeneity of the included studies and the absence of clinical data. Although this outcome showed greater consistency across studies, further standardization of experimental methodologies is needed.

### 4.1. Strengths and Limitations

A pre-registered PROSPERO protocol was followed. To achieve objectivity, both univariate and multivariate analyses were performed. This analysis is a novelty in the literature as no other meta-analysis has compared chitosan and sodium hypochlorite.

This study has some limitations: the study methods were not completely consistent in the articles.

### 4.2. Implications for Practice and Research

More clinical, in vitro, and animal studies are needed to provide a higher level of evidence, but our results suggest that chitosan is a promising agent as a root canal irrigation solution. It is essential that medical practices adopt new scientific results as soon as possible [50,51].

The measurement design that we propose methodologically facilitates more homogeneous results and clearer decisions for the clinician. In this regard, we suggest that further research should be conducted in the future using the same method, although the current results show that chitosan is an acceptable alternative to sodium hypochlorite.

## 5. Conclusions

Although chitosan demonstrated antimicrobial activity comparable to sodium hypochlorite in the included in vitro studies, this should not be interpreted as proof of clinical equivalence. At concentrations of chitosan above 1%, the antibacterial effect of chitosan becomes stronger compared to sodium hypochlorite; however, these findings should be interpreted with caution because the studies involved differences in chitosan concentration, formulation, and irrigation protocols. The present findings indicate similar laboratory performance under specific experimental conditions rather than interchangeable clinical effectiveness.

## Figures and Tables

**Table 1:** Heterogeneity.

Variable	Observed Variability Across Included Studies	How This May Affect Antimicrobial Efficacy/Interpretation
Chitosan concentration	0.1–2%	Concentration can alter protonated amino group availability, charge density, viscosity, and diffusion through dentin/biofilm.
Molecular weight	Clearly reported in Wang (150 kDa); qualitatively reported in Camacho-Alonso as low molecular weight; NR in most other studies	MW can influence solubility, diffusion into biofilm/dental tubules, and surface interaction with bacteria.
DDA/substitution degree	Wang reports substitution degree 85%; Shenoi reports 90% acetylation/deacetylation ambiguously; NR in most studies	DDA affects cationic charge density and antimicrobial interaction with bacterial cell walls.
Formulation	Solution, nanoparticles, HTCC derivative, chitosan-EDTA combinations, chitosan-CHX combinations, PDT/chitosan and laser adjunct protocols	Different formulations cannot be assumed equivalent; combinations may reflect synergy or adjunctive effects rather than chitosan alone.
Solvent/vehicle	1% acetic acid, 0.1% acetic acid, acidified water, DDW/PBS dilution, NR	Solvent affects chitosan solubility and protonation; Wang suggests DDW vs PBS changes antibacterial effect.
NaOCl concentration	2–5.2%	NaOCl efficacy is concentration- and contact-time-dependent; comparator strength differs by study.
Exposure time	3 min, 5 min, 10 min, 15–60 min, 24 h, 24/48/72 h agar readings; some studies NR	Longer contact increases killing; agar incubation time is not directly comparable with intracranial irrigation time.
Biofilm maturity/model	No biofilm agar diffusion; 48 h infected root canals; 7-day biofilms; 4-week and 6-week dental/root-canal biofilms	Mature dental biofilms are more resistant than planktonic/agar models; clinical relevance varies.
Reporting quality	MW and DDA/substitution degree absent or unclear in most studies; some contact times/suppliers also absent	Missing descriptors limit reproducibility and prevent robust moderator/subgroup analyses.

## Data Availability

The data that support the findings of this study are available from the corresponding author upon reasonable request.

## Supplementary Materials

The following supporting information can be downloaded at: https://www.mdpi.com/article/10.3390/medicina62081491/s1, Table S1: Methodological and physicochemical characteristics of the included in vitro studies evaluating chitosan-based irrigants against *Enterococcus faecalis* [4,9,10,11,12,26,27,28,29,30]; Table S2: Certainty of evidence with GRADE-PRO tool; Table S3: Risk of bias analysis; Table S4: QUIN TOOL [4,9,10,11,12,26,27,28,29,30]; Table S5: PRISMA Checklist [19].

## Nomenclature

Root canal treatment	A dental procedure used to treat infection or damage inside a tooth. It involves removing the infected or inflamed pulp (the soft tissue within the tooth), cleaning and disinfecting the root canals, and then filling and sealing them to prevent further infection.
Smear Layer	A thin film of debris that forms on the surface of dentin and inside the root canal walls during instrumentation. It consists of organic and inorganic materials, including dentin particles, pulp tissue remnants, and bacteria. The smear layer can block dentinal tubules and may interfere with proper disinfection and sealing of the root canal, so it is often removed during endodontic treatment.
Polymer	A large molecule made up of repeating smaller units called monomers, which are chemically bonded together. Polymers can be natural (such as cellulose or proteins) or synthetic (such as plastics).
Amino Polysaccharide	A type of polysaccharide (complex carbohydrate) that contains amino groups as part of its molecular structure. These molecules often play important biological roles, such as forming structural components in organisms. An example is chitosan, derived from chitin, which is found in the shells of crustaceans and is used in biomedical and dental applications for its biocompatibility and antimicrobial properties.
Polycationic Polymers	Polymers that carry multiple positive charges along their molecular chains. These positive charges allow them to interact strongly with negatively charged surfaces, such as bacterial cell walls, dentin, or other polymers. In dentistry and medicine, polycationic polymers are often used for their antimicrobial, adhesive properties—for example, in disinfectants, drug delivery systems, or bio adhesive materials. Examples include polyethyleneimine (PEI) and chitosan.
EDTA (Ethylenediaminetetraacetic Acid)	A chelating agent commonly used in dentistry and endodontics to remove the inorganic component of the smear layer from root canal walls.
