# Supplementary material for: Antimicrobial Efficacy of Chitosan vs. Sodium Hypochlorite Against Enterococcus faecalis: A Systematic Review and Meta-Analysis
Source: Medicina (Kaunas). 2026 Aug 3;62(8):1491. doi: 10.3390/medicina62081491 (PMC13515110; doi:10.3390/medicina62081491)
Supplement: Supplementary file 1 [file medicina-62-01491-s001.zip › Supplementary Table S1.pdf]

Methodological and physicochemical characteristics of the included in vitro studies evaluating chitosan-based irrigants against Enterococcus faecalis.

Study	Aim	Chitosan concentration	MW	DDA	Formulation of chitosan	NaOCl concentration	Exposure time	Biofilm / model	Main finding relevant to review	Assessment
Camacho-Alonso et al., 2017	Evaluate antibacterial efficacy of PDT and chitosan against E. faecalis in infected root canals and assess enhancement of PDT by chitosan.	0,30%	Low molecular weight (qualitative)	NR	Low-viscosity chitosan solution; PDT + chitosan adjunct	2,50%	NaOCl: 5 min. Chitosan alone: 100 µL topical application; dwell time not explicitly stated. PDT+chitosan: methylene blue and chitosan left 3 min, then 60 s laser.	Enterococcus faecalis	PDT+chitosan yielded the lowest CFU/mL and lowest contaminated area; chitosan alone produced results in the same statistical group as NaOCl/PDT in the reported comparisons.	CFU
Pillai et al., 2018	Compare antibacterial efficacy of 4% NaOCl, 2% CHX, MTAD and 2% chitosan against E. faecalis.	2,00%	NR	NR	Chitosan polymer solution/irrigant	4,00%	Agar plates incubated 24 h at 37°C after loading irrigant into wells	Enterococcus faecalis	MTAD had the highest inhibition zone; 2% chitosan had greater inhibition than 4% NaOCl but less than CHX and MTAD.	IR
Bhullar et al., 2021	Analyze and compare efficacy of three endodontic irrigating solutions against E. faecalis.	0,20%	NR	NR	Chitosan solution	5,00%	Test solutions delivered after 6-week inoculation; exact contact time not specified	Enterococcus faecalis	Mean CFU values: NaOCl 0.590, chitosan 2.118, saline 90.184; NaOCl exhibited maximum efficacy and chitosan was better than saline.	CFU
Shenoi et al., 2016	Compare antimicrobial efficacy of BioPure MTAD, chitosan, 2% CHX and 3% NaOCl against E. faecalis.	0,20%	NR	Paper states '90% acetylation' for chitosan; unclear whether this means degree of deacetylation.	Chitosan on saturated discs	3,00%	Disc diffusion; plates incubated 24 h at 37°C	Enterococcus faecalis	BioPure MTAD showed largest inhibition zone. 1% chitosan was not significantly different from 3% NaOCl; 0.2% chitosan showed no inhibition zone.	IR
Geethapriya et al., 2016	Evaluate chitosan and chitosan-EDTA against E. faecalis dentinal biofilm and smear layer removal compared with NaOCl/EDTA.	1,20%	NR	NR	Chitosan solution; chitosan-EDTA combinations	5,20%	Time-kill: 0, 15, 30, 45, 60 min; SEM biofilm exposure 24 h; smear-layer final irrigation 5 min	Dentinal biofilm	Chitosan and chitosan-EDTA produced 3-log reduction in sessile E. faecalis at 15 min; 5.2% NaOCl showed 99.98% inhibition at 15 min; chitosan-EDTA 1:1 effective for smear-layer removal with less erosion than EDTA.	IR
Goel et al., 2022	Compare the antimicrobial efficacy of 0.2% chitosan, 3% sodium hypochlorite and 2% chlorhexidine against E. faecalis, alone and in conjunction with an 810-nm diode laser.	0,20%	NR	NR	Chitosan solution; tested alone and with diode laser adjunct/activation	3,00%	10 mL irrigant total; 2 mL left in canal for 2 min and repeated 5 times (10 min total). Laser subgroups used 5-s irradiation repeated 5 times.	Enterococcus faecalis	2% CHX produced no growth; 0.2% chitosan showed lower CFU/mL than 3% NaOCl and saline. Diode laser after irrigation further reduced CFU/mL compared with irrigant alone or laser alone.	CFU
Jaiswal et al., 2017	Evaluate antibacterial efficacy of chitosan, CHX, propolis and NaOCl on E. faecalis biofilm.	0,20%	NR	NR	Chitosan solution; chitosan-CHX combinations	5,00%	3 mL irrigant for 10 min	Enterococcus faecalis	Chitosan-CHX combinations, CHX and NaOCl showed no statistically significant differences; 0.2% chitosan showed antibacterial activity but was less effective than the strongest groups.	CFU
Yadav et al., 2017	Evaluate antibacterial/antifungal efficacy and cytotoxicity of chitosan as an endodontic irrigant against E. faecalis and C. albicans biofilm on tooth substrate.	0,25%	NR	NR	Chitosan solution	3,00%	Biofilm assay: 1 mL irrigant for 10 min; paper-point sampling for 60 s. Agar/CFU assays also used.	Tooth substrate biofilm	Chitosan groups significantly reduced CFU and were reported to be at par with 3% NaOCl and 2% CHX; chitosan showed no cytotoxicity at 3 mg/mL and 10% cytotoxicity at 6 mg/mL.	CFU,IR
Janawathi et al., 2022	Evaluate antimicrobial efficacy of NaOCl, SDF and chitosan nanoparticles against E. faecalis as root canal irrigants.	0,10%	NR	NR	Chitosan nanoparticles (CNPs)	5,00%	0.25 mL irrigant on blotting paper; 2 h pre-diffusion; incubation/readings at 24, 48 and 72 h	Enterococcus faecalis	NaOCl showed the greatest inhibition zone, followed by SDF; CNPs were least efficacious among tested irrigants.	IR
Wang et al., 2020	Assess antibacterial effect of chitosan and N-(2-hydroxyl) propyl-3-trimethyl ammonium chitosan chloride (HTCC) on E. faecalis in planktonic and biofilm states.	0,25%	150 kDa	Substitution degree 85% reported; DDA not separately stated	Chitosan solution and HTCC derivative	2,00%	Planktonic CFU assay: 24 h. Biofilm on coverslips/dentine blocks: 24 h treatment after 7-day biofilm growth.	Enterococcus faecalis	Final MBCs in DDW were 70 µg/mL for chitosan and 140 µg/mL for HTCC. In biofilm experiments, 78 µg/mL chitosan in DDW nearly approached 2% NaClO effect.	CFU
