# Supplementary material for: Antimicrobial Efficacy of Chitosan vs. Sodium Hypochlorite Against Enterococcus faecalis: A Systematic Review and Meta-Analysis
Source: Medicina (Kaunas). 2026 Aug 3;62(8):1491. doi: 10.3390/medicina62081491 (PMC13515110; doi:10.3390/medicina62081491)
Supplement: Supplementary file 1 [file medicina-62-01491-s001.zip › Supplementary Table S2.pdf]

Supplementary Table S2. Certainty of evidence with GRADE-PRO tool

Question: Chitosan compared to sodium-hypochlorite for eliminating Enterococcus faecalis

Certainty assessment							№ of patients		Effect		Certainty	Importance
№ of studies	Study design	Risk of bias	Inconsistency	Indirectness	Imprecision	Other considerations	chitosan	sodium-hypochlorite	Relative (95% CI)	Absolute (95% CI)		
**colony forming unit (assessed with: CFU/ml)**												
6	randomised trials	not serious	not serious	not serious	not serious		94	94	-	MD **-0.28** (-1.54 lower to 0.97 higher)	high	CRITICAL
**inhibition rate (assessed with inhibition zone in mm)**												
6	randomised trials	not serious	not serious	not serious	not serious		60	60	-	MD **-4.12** (-12.25 lower to 4.01 higher)	high	CRITICAL

CI: confidence interval; **MD:** mean difference
