# Supplementary material for: Antimicrobial Efficacy of Chitosan vs. Sodium Hypochlorite Against Enterococcus faecalis: A Systematic Review and Meta-Analysis
Source: Medicina (Kaunas). 2026 Aug 3;62(8):1491. doi: 10.3390/medicina62081491 (PMC13515110; doi:10.3390/medicina62081491)
Supplement: Supplementary file 1 [file medicina-62-01491-s001.zip › Supplementary Table S3.pdf]

**Supplementary Table S3. Risk of bias analysis**

| <u>Unique ID</u> | <u>Study ID</u> | <u>Experimental</u> | <u>Comparator</u> | <u>Outcome</u> | <u>Weight</u> | <u>D1</u> | <u>D2</u> | <u>D3</u> | <u>D4</u> | <u>D5</u> | <u>Overall</u> |
|---|---|---|---|---|---|---|---|---|---|---|---|
| **Camacho Alonso** | **CFU** | chitosan | sodium-hypochlorite | number of bacteria | 0.175 | ● | ● | ● | ● | ● | ● |
| **Wang** | **CFU** | chitosan | sodium-hypochlorite | number of bacteria | 0.176 | ● | ● | ● | ● | ● | ● |
| **Yadav** | **CFU** | chitosan | sodium-hypochlorite | number of bacteria | 0.166 | ● | ● | ● | ● | ● | ● |
| **Goel** | **CFU** | chitosan | sodium-hypochlorite | number of bacteria | 0.152 | ● | ● | ● | ● | ● | ● |
| **Bhullar** | **CFU** | chitosan | sodium-hypochlorite | number of bacteria | 0.165 | ● | ● | ● | ● | ● | ● |
| **Jaishwal** | **CFU** | chitosan | sodium-hypochlorite | number of bacteria | 0.166 | ● | ● | ● | ● | ● | ● |
| **Yadav IR** | **IR** | chitosan | sodium-hypochlorite | inhibition rate | 0.166 | ● | ● | ● | ● | ● | ● |
| **Shenoi** | **IR** | chitosan | sodium-hypochlorite | inhibition rate | 0.167 | ● | ● | ● | ● | ● | ● |
| **Pillai** | **IR** | chitosan | sodium-hypochlorite | inhibition rate | 0.168 | ● | ● | ● | ● | ● | ● |
| **Janavathi** | **IR** | chitosan | sodium-hypochlorite | inhibition rate | 0.167 | ● | ● | ● | ● | ● | ● |
| **Geethapriya** | **IR** | chitosan | sodium-hypochlorite | inhibition rate | 0.168 | ● | ● | ● | ● | ● | ● |

*(D1) Bias arising from the randomization process.*
*(D2) Bias due to deviations from intended interventions.*
*(D3) Bias due to missing outcome data.*
*(D4) Bias in measurement of the outcome.*
*(D5) Bias in selection of the reported result.*
