# Supplementary material for: Antimicrobial Efficacy of Chitosan vs. Sodium Hypochlorite Against Enterococcus faecalis: A Systematic Review and Meta-Analysis
Source: Medicina (Kaunas). 2026 Aug 3;62(8):1491. doi: 10.3390/medicina62081491 (PMC13515110; doi:10.3390/medicina62081491)
Supplement: Supplementary file 1 [file medicina-62-01491-s001.zip › Supplementary Table S4.pdf]

Supplementary Table S4. QUIN TOOL

	Clearly stated aims/objectives	Detailed explanation of sample size calculation	Detailed explanation of sampling technique	Details of comparison group	Detailed explanation of methodology	Operator details	Randomization	Method of measurement of outcome	Outcome assessor details	Blinding	Statistical analysis	Presentation of results	**Sum**
Camacho-Alonso 2017	2	0	2	2	2	2	2	2	2	not applicable	2	2	20
Goel 2022	2	0	2	2	2	2	2	2	2	not applicable	2	2	20
Wang 2020	2	0	2	2	2	2	2	2	2	not applicable	2	2	20
Yadav 2017	2	0	2	2	2	2	2	2	2	not applicable	2	2	20
Bhullar 2020	2	0	2	2	2	2	2	2	2	not applicable	2	2	20
Jaishwal 2017	2	0	2	2	2	2	2	2	2	not applicable	2	2	20
Janawathi 2022	2	2	2	2	2	2	2	2	2	not applicable	2	2	22
Shenoi 2016	2	0	2	2	2	2	2	2	2	not applicable	2	2	20
Pillai 2018	2	0	2	2	2	2	2	2	2	not applicable	2	2	20
Geethapriya 2016	2	0	2	2	2	2	2	2	2	not applicable	2	2	20
